# The Co-occurrence of Self-Harm and Aggression: A Cognitive-Emotional Model of Dual-Harm

**DOI:** 10.3389/fpsyg.2021.586135

**Published:** 2021-02-25

**Authors:** Matina Shafti, Peter James Taylor, Andrew Forrester, Daniel Pratt

**Affiliations:** ^1^Division of Psychology and Mental Health, School of Health Sciences, Manchester Academic Health Science Centre, The University of Manchester, Manchester, United Kingdom; ^2^Division of Psychological Medicine and Clinical Neuroscienecs, School of Medicine, Cardiff University, Cardiff, United Kingdom

**Keywords:** dual-harm, co-occurrence, Suicide, self-injury, self-harm, assault, violence, aggression

## Abstract

There is growing evidence that some individuals engage in both self-harm and aggression during the course of their lifetime. The co-occurrence of self-harm and aggression is termed dual-harm. Individuals who engage in dual-harm may represent a high-risk group with unique characteristics and pattern of harmful behaviours. Nevertheless, there is an absence of clinical guidelines for the treatment and prevention of dual-harm and a lack of agreed theoretical framework that accounts for why people may engage in this behaviour. The present work aimed to address this gap in the literature by providing a narrative review of previous research of self-harm, aggression and dual-harm, and through doing so, presenting an evidence-based theory of dual-harm – the cognitive-emotional model of dual-harm. This model draws from previous studies and theories, including the General Aggression Model, diathesis-stress models and emotional dysregulation theories. The cognitive-emotional model highlights the potential distal, proximal and feedback processes of dual-harm, the role of personality style and the possible emotional regulation and interpersonal functions of this behaviour. In line with our theory, various clinical and research implications for dual-harm are suggested, including hypotheses to be tested by future studies.

## Introduction

Self-harm is an umbrella term that encompasses both suicidal behaviours (self-injury behaviour with intent to end one’s life) and non-suicidal self-injury (NSSI; self-injury without intent to die). There is much debate in the literature as to whether it is meaningful to make a distinction between suicidal and non-suicidal forms of self-injury (Butler and Malone, 2013; Kapur et al., 2013). As with self-harm, aggression is variably defined within the literature. Aggressive behaviour may range in severity from minor acts (e.g., verbal aggression) to more serious acts (e.g., stabbing and killing).

While self-harm and aggression may initially seem distinct, research has consistently shown that these behaviours co-occur across various populations. The co-occurrence of self-harm and aggression during the course of an individual’s lifetime has been termed “dual-harm” (Slade, 2019). There is emerging evidence to suggest that, compared to those who engage in self-harm alone or aggression alone (“sole-harm”), individuals who dual-harm may have distinct characteristics. These include greater levels of contextual and personal risk, and a riskier pattern of harmful behaviours (Bortolato et al., 2013; Tang et al., 2013; O’Donnell et al., 2015; Terzi et al., 2017; Harford et al., 2018; Kottler et al., 2018; Slade, 2018; Richmond-Rakerd et al., 2019; Steeg et al., 2019; Carr et al., 2020; Slade et al., 2020). Such evidence has led researchers to hypothesise that, rather than self-harm and aggression simply co-occurring, dual-harm may be an independent construct that stands separate from sole-harm behaviour.

Despite empirical support for dual-harm, little research has investigated this construct. At the time of writing, there is a lack of an agreed theory that explains why individuals may engage in both self-harm and aggression. Given the high-risk profile shown by those who dual-harm, it is important that we develop our theoretical understanding of this behaviour. Doing so may provide support for considering dual-harm as a unique and independent clinically valid entity.

The present article aims to address the gaps in the literature by presenting a theoretical model of dual-harm, focusing on the cognitive and emotional aspects of this behaviour. First, we will provide a narrative review of previous research of self-harm, aggression and dual-harm, with a particular focus on psychological factors. Subsequently, the paper will draw from this review to propose a cognitive-emotional model of dual-harm that accounts for why individuals may engage in both aggression and self-harm during the course of their lifetime.

To provide a comprehensive review of self-injury behaviour, and given that much research has not identified suicidal intent, the present paper will draw from the broader self-harm literature (i.e., self-injury irrespective of intent to end life). Furthermore, we will define aggression according to its most common definition within social psychology and aggression research: “any behaviour directed toward another individual that is carried out with the proximate (immediate) intent to cause harm…the perpetrator must believe that the behaviour will harm the target, and that the target is motivated to avoid the behaviour” (Anderson and Bushman, 2002, p. 28). The above definition encapsulates all forms of aggression, regardless of severity of intent. This definition will be adopted given its demonstrated utility in the development and testing of aggression theories, as well as evidence that similarly defined behaviours have comparable aetiologies (Allen and Anderson, 2017). Our work will inform future research of dual-harm by providing testable hypotheses for further investigation and therefore, help to extend our understanding of this behaviour.

## Self-Harm

### Emotional Regulation

Emotional dysregulation has gained great support as a core component of self-harm. This construct has been defined differently across the literature, reflecting its various conceptualisations. Our paper will adopt Gratz and Roemer’s (2004) definition which highlights the functionality of all emotions. According to Gratz and Roemer, emotional regulation is the “(a) awareness and understanding of emotions, (b) acceptance of emotions, (c) ability to control impulsive behaviours and behave in accordance with desired goals when experiencing negative emotions, and (d) ability to use situationally appropriate emotional regulation strategies flexibly to modulate emotional responses as desired in order to meet individual goals and situational demands” (Gratz and Roemer, 2004, p. 42). The absence of any of these components would indicate emotional dysregulation (Gratz and Roemer, 2004). The above definition has been shown to be clinically useful, with research demonstrating a relationship between the emotional regulation components and harmful behaviours (Gratz, 2007; Peh et al., 2017; Velotti et al., 2020; Yeo et al., 2020).

Studies have consistently found that individuals who engage in self-harm have greater levels of emotional dysregulation compared to those who do not (Weiss et al., 2015; Taylor et al., 2018). Taylor et al. (2018) conducted a meta-analysis of 46 studies investigating the functions of NSSI, including emotional regulation. The review found that emotional regulation was the most common function of NSSI, with 63–78% of participants reporting it as the function of their behaviour. The role of emotional dysregulation in self-harm has been demonstrated across community, clinical and forensic samples, providing strong support for the generalisability of findings (Dixon-Gordon et al., 2012; Andover and Morris, 2014). For example, Borderline Personality Disorder (BPD) is characterised by instability in interpersonal functioning, cognitions, affect, and impulsivity (American Psychiatric Association, 2000). It has been suggested that emotional regulation is a central component of self-harm in BPD (Putnam and Silk, 2005). The above hypothesis may be supported by findings that interventions targeting emotional regulation in BPD reduce the frequency of self-harm (Rizvi et al., 2016; Sahlin et al., 2017a; Wetterborg et al., 2020). Such research suggests that emotional dysregulation may be a key causal pathway underlying self-harm in those with psychopathology.

**FIGURE 1:**
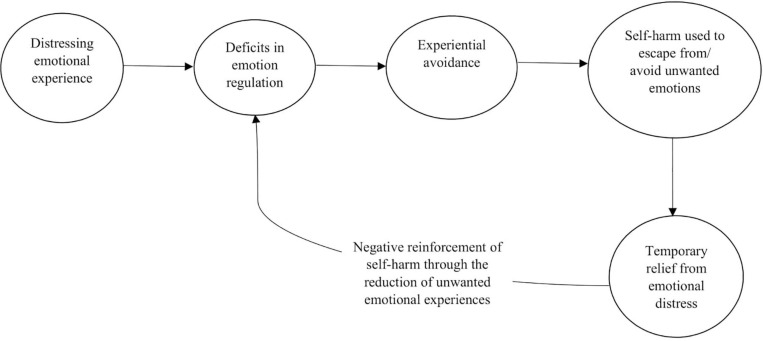
Experiential avoidance theory of self-harm. This figure provides a simplified illustration of the experiential avoidance theory of self-harm. As highlighted, deficits in emotional regulation may contribute to an individual using experiential avoidance as a response to distressing emotional experiences. Here, experiential avoidance refers to attempts to avoid distressing emotions, even when such attempts may cause harm to the individual. According to the theory, the individual may use self-harm as an experiential avoidance strategy to escape from or avoid their unwanted emotions. Self-harm may provide the individual with temporary relief from their emotional distress, thus leading to negative reinforcement of their self-harm. This negative reinforcement feeds back into the person’s emotional regulation deficits, thus repeating the cycle of experimental avoidance and maintaining the use of self-harm.

Given the strong evidence, the most recognised theories of self-harm highlight emotional regulation as the core function of this behaviour (Hasking et al., 2017). The exact mechanism of how emotional regulation operates in self-harm varies across theories. For example, Hasking et al. (2017) suggested a cognitive-emotional model of NSSI, where individuals with emotional dysregulation and maladaptive cognitions are more likely to use NSSI to modulate emotionally negative situations. Alternatively, Chapman et al.’s (2006) experiential avoidance theory (Figure 1) suggests that emotional dysregulation, combined with a negative emotional experience, may lead to the use of self-harm as temporary relief from undesired situations or emotions. This relief may reinforce self-harm behaviour, causing the development of self-harm into a repeated classical conditioned response to negative emotions. While the exact processes in the above theories may vary, their shared conclusion remains: emotional regulation is a key function of self-harm, used as a response to negative feelings.

### Adverse Events

Research has provided evidence for the role of early environmental mechanisms in self-harm. These include bullying, familial dysfunction and peer rejection, and most notably, negative childhood experiences within the family, such as emotional neglect and abuse (Fliege et al., 2009; Di Pierro et al., 2012; Swannell et al., 2012; Esposito et al., 2019). For example, Fliege et al. (2009) systematically reviewed 59 studies examining the distal and proximal risk-factors of NSSI. The most frequently reported risk-factor was childhood trauma, including emotional neglect and psychological and physical abuse.

To account for how early adverse factors may lead to self-harm, diathesis-stress models suggest that they interact with biological factors to develop certain personality traits and cognitive styles, including impaired self-regulation and decision-making (Brodsky, 2016). These increase an individual’s risk of engaging in self-harm in response to a stressor (Brodsky, 2016). On the other hand, the Interpersonal Theory of Suicide (Joiner, 2007; Van Orden et al., 2010) proposes that the desire to engage in suicidal behaviours occurs when an individual experiences perceived burdensomeness and thwarted belongingness. An individual acts on this desire when they have the capability to engage in suicidal behaviour. It has been suggested that adverse childhood events (e.g., physical or sexual abuse) may lead to both the desire and capability to engage in suicidal behaviour, therefore increasing the risk of self-harm (Van Orden et al., 2010).

### Interpersonal Functions

Although intrapersonal functions, such as emotional regulation, are the most frequently reported reason for NSSI (Klonsky, 2009; Saraff and Pepper, 2014; Taylor et al., 2018), there is evidence that this harmful behaviour may also serve interpersonal functions (e.g., establishing autonomy, communicating distress; Hilt et al., 2008; Muehlenkamp et al., 2013; Sadeh et al., 2014; Gardner et al., 2016; Taylor et al., 2018). For example, Gardner et al. (2016) examined NSSI in offenders and found that 73% of individuals reported an interpersonal function for their NSSI, such as creating a boundary from others and seeking help. Such findings highlight that theories should consider the interpersonal and intrapersonal motivations of self-harm, as well as the social context in which they occur.

## Aggression

### Adverse Events

As with self-harm, a range of environmental factors have been associated with aggression (Mendes et al., 2009). Mendes et al.’s (2009) systematic review revealed that familial dysfunction, poverty, family criminality, and educational underachievement are significant risk-factors for aggression. Studies have particularly provided support for the role of negative childhood experiences within the family, such as abuse, harsh discipline and early neglect, in aggression (Lansford et al., 2007; Duke et al., 2010; Topitzes et al., 2012; Milaniak and Widom, 2015). Duke et al.’s (2010) prospective study of 135,549 students found that early adverse experiences (e.g., physical abuse, sexual abuse, and household dysfunction) were significantly associated with aggression in adolescence. For each type of adverse event reported by participants, the estimated risk of violence increased from 35 to 144% (Duke et al., 2010).

Numerous processes, in particular, biological mechanisms, have been proposed to underlie the pathway from early aversive experiences to aggression. These experiences have been suggested to interact with genetic predisposition to increase an individual’s vulnerability to aggression (Byrd and Manuck, 2014). For example, the MAOA gene has been found to moderate the influence of childhood maltreatment on aggression, suggesting that gene-environment interactions play a role in this behaviour (Byrd and Manuck, 2014). Furthermore, diathesis-stress models suggest that early adverse events interact with genetic mechanisms to develop an antisocial personality style. This personality style increases an individual’s risk of using aggression in response to a stressor (Ferguson et al., 2008). As well as influencing personality, researchers have highlighted the effect of negative childhood experiences on emotional functioning. Such experiences have been shown to be significantly associated with impairments in emotional processes, including emotional regulation, emotional reactivity and emotion recognition (Pechtel and Pizzagalli, 2011). These impairments may increase the likelihood of engaging in aggression in response to stressful stimuli (Fox et al., 2015).

### Personality

Personality traits, including emotional reactivity, impulsivity and neuroticism, have been significantly linked to aggression (Ramirez and Andreu, 2006; Jones et al., 2011). Psychopathy is a personality style characterised by interpersonal, affective, behavioural, and antisocial characteristics, as well as a disregard for other people’s rights and societal norms (Hare, 1996). Psychopathy has been found to be one of the strongest dispositional factors associated with aggression, including its most stable and violent patterns, across community, clinical and forensic populations (Neumann and Hare, 2008; Forsman et al., 2010; Blais et al., 2014; McCuish et al., 2015; Gray and Snowden, 2016). A meta-analysis of 53 studies found that psychopathy was significantly associated with instrumental and reactive violence, with moderate effect sizes (*r* = 0.36, *r* = 0.35, respectively; Blais et al., 2014). Furthermore, biological studies have demonstrated that individuals with psychopathy show unique neurobiological patterns associated with persistent aggression, including differences in their brain’s function and structure (Gregory et al., 2012). Given the consistent evidence for psychopathy as a key mechanism for aggression, measures of this personality are often utilised in risk-assessments for violence and recidivism within forensic settings (Viljoen et al., 2010).

### Emotional Regulation

Emotional regulation has been argued to be a function of aggression, with significant positive associations reported between emotional dysregulation and aggression amongst adults and adolescents (Davidson et al., 2000; Cohn et al., 2010; Roberton et al., 2012). Moreover, individuals who engage in aggression have been found to show differences in their brain’s central circuitry that is responsible for emotional regulation (Davidson et al., 2000). Roberton et al. (2012) suggested that emotional regulation may lead to aggression due to either under-regulation or over-regulation. Under-regulation refers to when an individual fails to sufficiently contain their difficult emotional experience and prevent impulsive behaviours. Such under-regulation may occur through increased negative affect and physiological arousal, decreased inhibitions against harmful behaviours and impaired decision-making, causing the individual to be aggressive. Alternatively, over-regulation occurs when regulation strategies are used to stop an emotional experience from occurring. An individual may use aggression to suppress and avoid their own emotional experience by directing harm toward others.

Individuals with psychopathy have been shown to have high levels of emotional dysregulation (Casey et al., 2013; Donahue et al., 2014; Garofalo et al., 2018). Donahue et al. (2014) investigated emotional regulation in 119 adults, comprised of undergraduate students and offenders referred to outpatient anger management programmes. Psychopathy scores in participants were significantly associated with subscales within the Difficulties in Emotion Regulation Scale (*r* = 0.30, *p* < 0.01), including non-acceptance of emotions (*r* = 0.23), impulse control difficulties (*r* = 0.35) and lack of emotional clarity (*r* = 0.26). Such findings highlight the importance of investigating the position of emotional dysregulation upon the causal pathway to aggression in personality styles such as psychopathy.

### General Aggression Model

**FIGURE 2:**
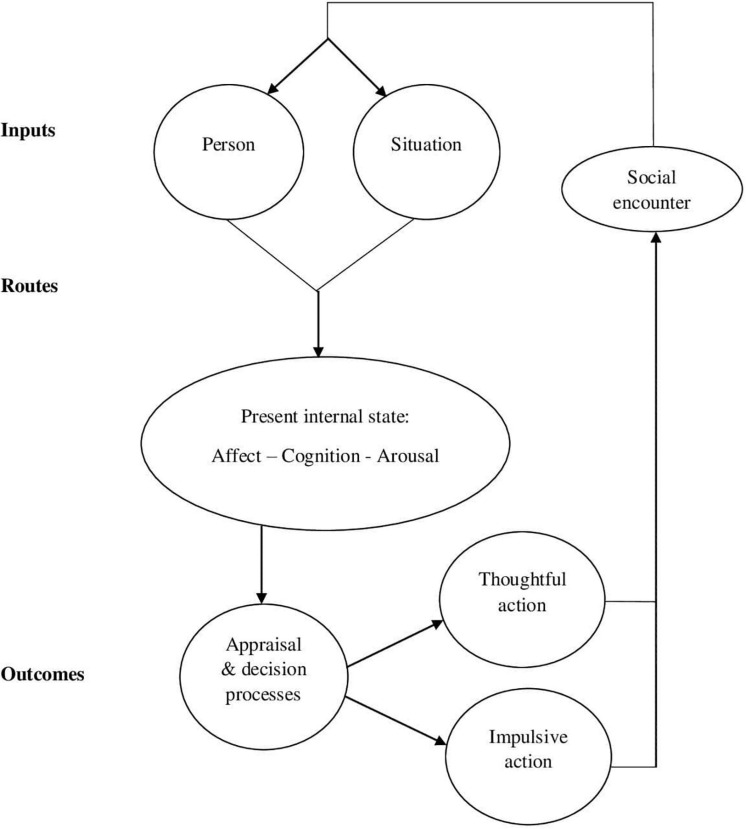
General Aggression Model. This figure highlights the proximal process of aggression in the General Aggression Model. The proximal process occurs in three stages: Inputs, routes, and outcomes. First, in the input stage, person-related factors and situations act as inputs which, in the routes stage, activate certain emotions, cognitions and arousals in the individual. The person’s internal state then affects appraisal and decision-making processes in the outcome stage, leading to them engaging in either a thoughtful action or impulsive action, such as aggression. This behavioural outcome impacts the individual’s social encounter and feeds back into person and situation related factors through learning mechanisms, thus perpetuating the model’s process.

One of the most widely accepted theories of aggression is the General Aggression Model (GAM; 15; Figure 2). The GAM is predominantly a social cognitive theory that divides the pathway to aggression into two processes: proximal factors (i.e., those that operate in the current state) and distal factors (i.e., those that occur over a long period of time). Distal factors, specifically, biological and environmental modifiers, combine to influence proximal factors. These proximal factors then lead to aggression through three stages: inputs, routes, and outcomes. These stages highlight the role of personal and situational related factors, as well as emotional and cognitive processes. The GAM highlights that the behavioural outcome of aggression impacts the environmental response and feeds back into the person and situation related factors through learning mechanisms, thus perpetuating the model’s process (Anderson and Bushman, 2002). According to the model, each stage may be considered a learning trial in which aggression-related knowledge structures are reinforced.

Although there is wide support for the GAM, the model has been subject to criticism (Ferguson and Dyck, 2012). The GAM focuses on the role of cognitive processes in aggression, in particular knowledge structures (Anderson and Bushman, 2002). Whilst the model includes the role of evidence-based biological, environmental, social, affective, and personality related factors, these are largely discussed in relation to how they link to cognitive mechanisms. Consequently, the GAM may be considered to be an inadequate or incomplete account as it views aggression through a particular lens. To provide a comprehensive framework for aggression, a more integrated understanding of this behaviour may be required.

### Diathesis-Stress Theories

Diathesis-stress theories of aggression may address some limitations of the GAM. These theories emphasise that individual differences in biological, personality and environmental related factors combine to lead to aggression. For example, the Catalyst Model (Ferguson et al., 2008) proposes that if an individual has been exposed to early adverse environmental factors, their genetic predisposition to aggression is more likely to lead to an aggressive personality style. The Catalyst Model highlights that individuals with such personality styles are then more likely to engage in aggression when they experience environmental stress. Support for the Catalyst model is provided by evidence for the role of stress and gene × environmental interaction effects on aggression, as well as findings that this model is a stronger predictor of aggression than the GAM (Ferguson et al., 2008; Ferguson and Dyck, 2012).

### Social Determinants

Despite evidence for the diathesis-stress model and GAM, these theories do not elaborate on the specific social contextual mechanisms that may provoke or minimise aggression. Situational and social contextual factors, such as social threat, social identity, peer status, and nature of the perpetrator’s and target’s relationship, have been found to be associated with aggression (Prinstein and Cillessen, 2003; Goldstein and Tisak, 2004; Coccaro et al., 2007; Richardson and Hammock, 2007; Faris and Ennett, 2012; Graham et al., 2013). Given such findings, researchers have highlighted that aggression may be interpersonally motivated and it is imperative that we consider this behaviour in the social context within which it is perpetrated (Prinstein and Cillessen, 2003; Richardson and Hammock, 2007).

## Dual-Harm

Research has widely distinguished self-harm from aggression, approaching these behaviours as two distinct constructs. Such separation may be a reflection of the contrasting perceptions surrounding harmful behaviours. Aggression is often seen as an unreasonable act in which an individual offends against others, consequently leading to a reactive response, typically in the form of containment and punishment orientated strategies (Slade, 2019). In contrast, self-harm is perceived as a sign of distress and an act against the self, which is more likely to elicit a care-giving response (Slade, 2019). Despite their historic separation, there is increasing evidence that self-harm and aggression are linked and co-occur. Furthermore, research has found that these behaviours are associated with common risk-factors, such as negative childhood experiences, impulsivity, impairments in emotional functioning, and genes related to dysfunctional serotonergic systems (Boxer, 2010; Bortolato et al., 2013; O’Donnell et al., 2015; Jordan and Samuelson, 2016; Sahlin et al., 2017b; Terzi et al., 2017).

The co-occurrence of self-harm and aggression, and the link between these two behaviours, has consistently been shown within community, clinical, forensic, adult, and adolescent samples. O’Donnell et al.’s (2015) systematic review of 23 studies found that the prevalence of aggression in those who had self-harmed exceeded 20% in most studies, with the highest reported prevalence rate being 74%. Moreover, in 23 studies that examined the association between harmful behaviours, most reported a significant positive correlation between self-harm and aggression (*r* = 0.12–0.62). The researchers also reviewed 24 studies that had not selected their sample for either harmful behaviour. In most studies, the prevalence rate of co-occurring self-harm and aggression exceeded 15%, with the highest prevalence rate being 47%. Furthermore, individuals who engaged in one of the harmful behaviours were significantly more likely to engage in the other behaviour (*odds ratio =* 1.05–38.55). Given that O’Donnell et al. (2015) reviewed studies across various populations, settings, designs, measurements, and data, their findings suggest that self-harm and aggression co-occur and are linked independently of methodological differences.

Richmond-Rakerd et al.’s (2019) 20-year cohort study of 2,049 twins within the general population in the United Kingdom provided further support for the co-occurrence of self-harm and aggression. 4.7% of participants reported to have previously engaged in both self-harm and violent crime. Furthermore, the risk of committing a violent crime was more than three times greater in those who had engaged in self-harm, compared to those who had not (*odds ratio* = 3.50). This association remained significant when only police records (*odds ratio* = 3.26) and only self-reports of violent crime *(odds ratio* = 3.50) were used, suggesting that the association between self-harm and aggression is not simply a reflection of assessment methods.

To date, the largest population-based investigation of dual-harm is Sahlin et al.’s (2017b) longitudinal cohort study of 1,850,525 individuals from the general population. During the average follow-up time of 8.1 years, 0.4% of the total sample had been in contact with healthcare due to self-harm and convicted of a violent crime. Specifically, 14.8% of self-harming patients had previously been convicted of a violent crime. After adjusting for psychiatric comorbidity, this represented a two-fold risk of having a conviction of violent crime amongst self-harm patients, in comparison to those who had not been in contact with healthcare due to self-harm.

While aggression is present amongst a third of those who have engaged in self-harm in community samples (O’Donnell et al., 2015), this figure has been reported to rise to over half within clinical and forensic samples (Plutchik et al., 1989; Slade, 2018; Slade et al., 2020). Slade et al. (2020) investigated harmful behaviours in 965 male prisoners in England using official HM Prison Service data. Results revealed that 11% of prisoners had engaged in dual-harm. Furthermore, there was a significant positive correlation between self-harm and aggression (*r* = 0.258), with 60% of those engaging in self-harm, having also engaged in aggression. This represented an almost fourfold increased risk of aggression for those with a history of self-harm, compared to those who had not engaged in self-harm (*odds ratio* = 3.81, *p* < 0.001). In another study of 326 prisoners from two prisons in England, it was found that up to 42% of prisoners who had engaged in aggression, had also engaged in self-harm (Slade, 2018). Moreover, Daffern and Howells (2009) examined harmful behaviours in 41 patients within a high-security personality disorder hospital. Results revealed that 46% of patients engaged in dual-harm during their stay at the hospital. The above studies suggest that rather than engaging in sole-harm behaviour, many high-risk individuals within forensic and clinical populations will engage in dual-harm.

### A Unique Clinical Construct?

**FIGURE 3:**
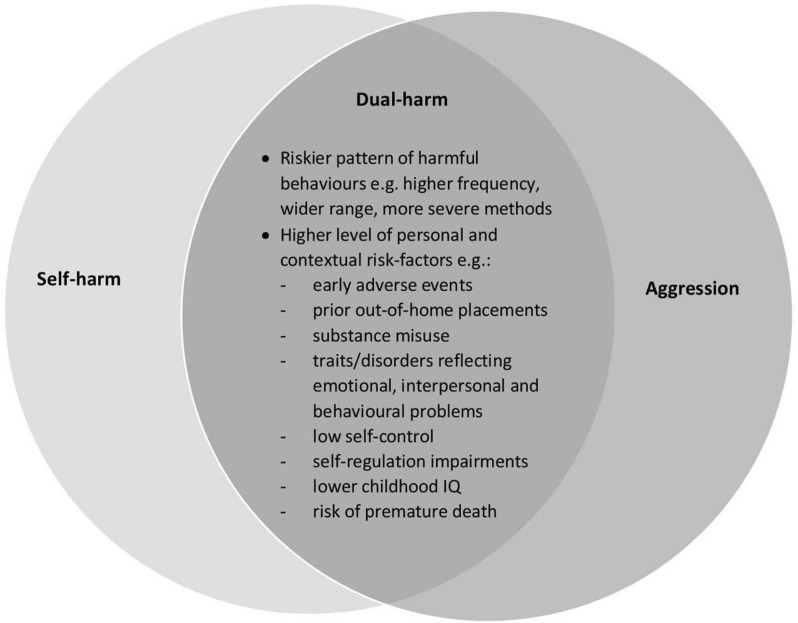
Potential distinct profile of dual-harm. This Venn diagram highlights the potential mechanisms that may distinguish dual-harm as a separate clinical entity from the behaviours of self-harm alone and aggression alone. According to previous research, compared to those who engage in sole-harmful behaviours, individuals who have a history of dual-harm are significantly more likely to engage in a higher frequency of harmful behaviours, as well as a wider range and more severe methods of risky behaviours. Furthermore, these individuals have been found to have higher levels of personal and contextual risk-factors. Such higher levels of risk in those who dual-harm may be an important factor that distinguishes dual-harm from the sole-harm behaviours of self-harm and aggression.

There is growing evidence that compared to those who engage in sole-harm, individuals who dual-harm may be distinguished by unique characteristics and show a riskier pattern of harmful behaviours (Figure 3). For example, Slade et al.’s studies (Slade, 2018; Slade et al., 2020) found that offenders with a history of dual-harm spent a significantly longer time in prison (on average, 40% longer) than those who had sole-harmed. This group also contributed to a higher rate and wider range of aversive prison incidents (e.g., arson and property damage) and were more likely to use a wider range and more lethal methods of self-harm (e.g., overdose).

Such unique characteristics have further been shown within community and clinical samples (Tang et al., 2013; O’Donnell et al., 2015; Harford et al., 2018; Carr et al., 2020). Boxer (2010) investigated dual-harm amongst 476 inpatients in a secure youth mental health service. Compared to the sole-harm group, the dual-harm group were significantly more likely to show personal and contextual risk across different factors, including physical, sexual and emotional abuse, neglect, age of aggression onset, prior out-of-home placements (e.g., hospitalisation and foster care), and emotional and behavioural disorders. Finally, the dual-harm group showed high continuity in their harmful behaviours from before treatment to during treatment, with 74% exhibiting both self-harm and aggression during treatment, and 97% showing either self-harm or aggression. Boxer (2010) outlined the potential importance of such findings, highlighting that harmful behaviours shown by those with a history of dual-harm may persist over time. As such, clinicians may be able to predict with greater certainty that individuals who enter mental health treatment with a history of dual-harm will likely engage in harmful behaviours during their stay.

Richmond-Rakerd et al.’s (2019) study demonstrated that individuals with a history of dual-harm may have a distinct personality style. Compared to those who had a history of sole-harm, individuals who had dual-harmed were significantly more likely to have traits relating to emotional and interpersonal liability (*d* = −0.15 to −0.06), as well as problems with self-control and self-regulation (*odds ratio* = 1.82). They were also more likely to have lower childhood IQ, which the researchers suggested indicates impairments in executive functioning. Additionally, the dual-harm group were more likely to have experiences of childhood maltreatment (*odds ratio* = 2.46) and adolescent victimisation (*odds ratio* = 2.40). As with Slade et al.’s (Slade, 2018; Slade et al., 2020) research, these individuals also demonstrated more lethal self-harm (e.g., hanging) and aggressive behaviours. The above studies are supported by findings that, in comparison to sole-harm, individuals who dual-harm are significantly more likely to exhibit traits reflecting emotional and interpersonal liability, self-regulation impairments, substance misuse disorders, greater risk of premature death (*incidence rate ratio* = 29.37), have experienced early adverse events, and show a more severe, frequent and wider range of harmful behaviours (Bortolato et al., 2013; Tang et al., 2013; O’Donnell et al., 2015; Terzi et al., 2017; Harford et al., 2018; Kottler et al., 2018; Slade, 2018; Steeg et al., 2019; Carr et al., 2020; Slade et al., 2020).

In the context of dual-harm, it may be that, rather than only co-occurring, self-harm and aggression possess common vulnerabilities, causal pathways and functionality. If this is the case, individuals who engage in harmful behaviours may be categorised into the following groups: *self-harm alone, aggression alone*, and *dual-harm*, each with a distinct risk-profile and patterns of behaviour. Accordingly, management of dual-harm may benefit from tailored approaches that address the distinct needs of this potentially unique high-risk group. Nevertheless, there is currently no national clinical guidelines for the prevention, management and treatment of dual-harm, reflected by the lack of literature in this area. The National Institute of Health and Care Excellence (NICE) provides separate guidance for aggression and self-harm, with no guidelines for those who engage in both behaviours (National Institute of Health Care Excellence, 2011; National Institute of Health Care Excellence, 2015). For example, within the NICE guideline for aggression (National Institute of Health Care Excellence, 2015), self-harm is only mentioned to highlight the lack of evidence of this behaviour as a risk-factor for aggression.

Although evidence points to the notion that those who dual-harm represent a unique high-risk group, we still have limited understanding of this behaviour. There is a need to investigate whether dual-harm should be considered and treated separately from sole-harm behaviours. Furthermore, previous literature has not offered a definition of dual-harm that specifies how close in time self-harm and aggression must co-occur (Slade, 2018, 2019; Slade et al., 2020). According to working definitions, an individual may self-harm and be aggressive at different points during their lifetime, and this would be considered dual-harm. However, should an individual who has repeatedly self-harmed and been aggressive throughout their lifetime be categorised in the same group as someone who has self-harmed once during adolescence and then been aggressive years later? It may be appropriate to suggest that self-harm and aggression should co-occur within a certain window of time for the behaviour to be considered as dual-harm. Research should explore the impact of adopting different criteria for the definition of dual-harm in order to identify a clinically useful assessment of this behaviour.

## A Cognitive-Emotional Model of Dual-Harm

**FIGURE 4:**
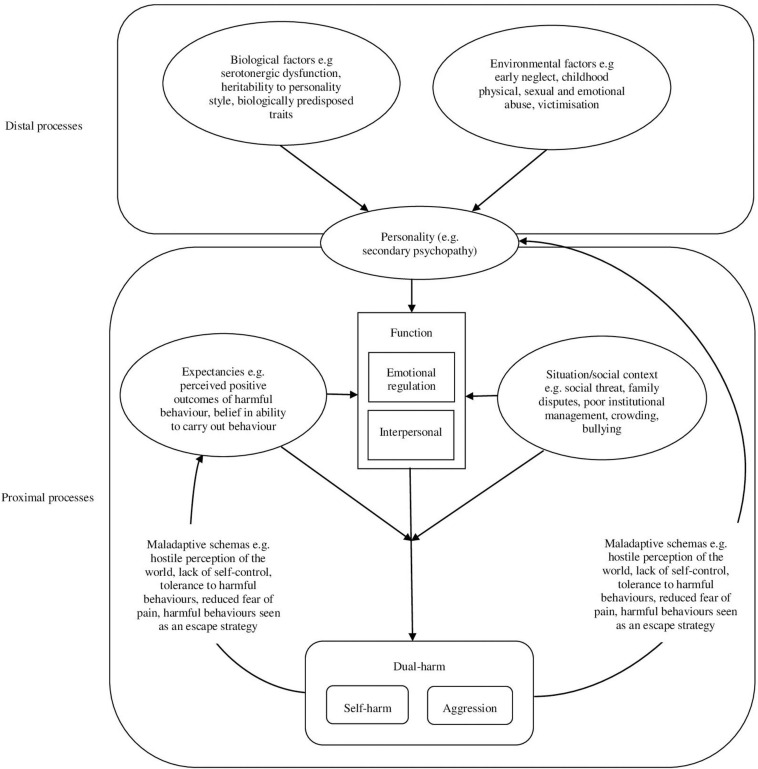
The cognitive-emotional model of dual-harm. This figure illustrates the cognitive-emotional model of dual-harm. Here, the causal pathways to dual-harm are divided into two main processes: distal and proximal. In the distal processes, biological and environmental factors combine to develop a certain personality style. Through its effects on cognition, arousal and affect, this personality style facilitates the proximal processes of dual-harm by predisposing the individual to harmful behaviours. In the proximal processes, the social context/situation the individual is in and their expectancies of harmful behaviours influences the function of their dual-harm behaviour (i.e., emotion regulation or interpersonal motivation), as well as the specific harmful behaviour that they choose to engage in (i.e., self-harm or aggression). Specifically, the individual may choose to engage in dual-harm as an emotional regulation response to their distressing negative emotions. Alternatively, they may choose to engage in dual-harm to fulfil an interpersonal function. Finally, the outcome behaviour affects the environmental response and individual’s experience. Through learning processes, this response may reinforce the individual’s maladaptive schemas and feed back into their personality traits and expectancies, thereby repeating the process of dual-harm.

Based on our narrative review of previous literature, we propose a cognitive-emotional model of dual-harm (Figure 4). This model draws from components of the GAM and diathesis-stress theories by highlighting the potential distal, proximal and feedback processes of dual-harm, as well as the key role of personality. Furthermore, we propose that emotional regulation and interpersonal motivations are the main functions of this behaviour. The different mechanisms of our cognitive-emotional model of dual-harm are summarised below.

### Distal Processes

Akin to the GAM, our model comprises two main processes: distal and proximal. While the distal processes indirectly inform a vulnerability toward dual-harm over a period of time, proximal processes more directly lead to the occurrence of this behaviour.

#### Personality

Personality-related factors, specifically, traits associated with emotional and interpersonal liability, have been shown to be distinguishing features in those who dual-harm (Richmond-Rakerd et al., 2019). Furthermore, research has shown that personality is associated with distinct levels of stress and coping strategies. For example, those with maladaptive personality traits, such as neuroticism, are more likely to use avoidant coping strategies (e.g., escape avoidance or self-blame), which may often lead to harmful behaviours (Afshar et al., 2015; Zainah et al., 2019). Individuals with maladaptive traits have also been shown to be more likely to experience stress and negative emotions (Vollrath and Torgersen, 2000; Zainah et al., 2019). Such findings suggest that an individual’s personality is linked to the level of emotional distress they experience, as well as the strategies they use to respond to such distress. Accordingly, in line with the GAM and diathesis-stress theories that highlight the important role of personal-related factors, our model proposes that a personality style that predisposes individuals to emotional and interpersonal liability, maladaptive coping strategies and harmful behaviours, may be a key distal component of dual-harm.

Secondary psychopathy may be a personality style that increases the likelihood of an individual engaging in dual-harm. This construct is a variant of psychopathy (the other being primary psychopathy), characterised by traits associated with an antisocial and unstable lifestyle (Hare, 2016). These include high impulsivity, poor anger control, sensation seeking, irresponsible behaviour, emotional instability, and antisocial behaviours (Hare, 2016). When conceptualised into its two variants, secondary psychopathy, but not primary psychopathy, has been linked to both self-harm and aggression (Douglas et al., 2006; Hicks et al., 2010; Smith et al., 2014; Pennington et al., 2015). Such findings may be attributed to the traits of secondary psychopathy that may make individuals more vulnerable to both harmful behaviours. For example, compared to primary psychopathy, individuals with secondary psychopathy have been found to have significantly higher levels of emotional distress, impulsivity, violent and criminal behaviour, mental health problems, substance abuse, victimisation, and poorer levels of behavioural control and interpersonal functioning (Skeem et al., 2007; Vaughn et al., 2009; Hicks et al., 2010). In our cognitive-emotional model, we suggest that personality style may be a key distal pathway to dual-harm. This is due to the emotional, cognitive and arousal characteristics of the personality, such as those of secondary psychopathy, which may form a predisposed vulnerability to both self-harm and aggression.

While we specify the role of secondary psychopathy, our model may be extended to other personality styles that are vulnerable to harmful behaviours and possess similar traits. For example, many characteristics of secondary psychopathy overlap with BPD symptoms, including impulsivity, emotional distress and low behavioural control. Research has also shown that these constructs are significantly linked to one another (Skeem et al., 2007; Miller et al., 2010). Therefore, our model may apply to other personality constructs, such as BPD, that relate to its various theoretical components.

#### Biological and Environmental Factors

Compared to those who sole-harm, individuals who engage in dual-harm have been found to be significantly more likely to have experienced early adverse life experiences (Richmond-Rakerd et al., 2019; Carr et al., 2020). Studies have also found that those with a history of dual-harm engaged in their first act of harmful behaviour earlier than those who engaged in sole-harm (Slade et al., 2020). These findings have been attributed to the notion that initiation of harmful behaviours may begin at an earlier stage for those who dual-harm, given that they are more likely to have experienced early adverse life events (Slade et al., 2020).

In consideration of such findings, our cognitive-emotional model of dual-harm suggests that in addition to personality, biological and environmental factors may also underpin the distal process of this behaviour. Similar to the GAM and diathesis-stress theories, we propose that adverse environmental factors, notably negative childhood experiences, interact with predisposed biological factors to develop a personality style that is vulnerable to dual-harm. Support for the above notion may be provided by research that has shown that a combination of early aversive environmental and genetic factors interact to develop harmful behaviours and personality style, including secondary psychopathy (Bakermans-Kranenburg and Van Ijzendoorn, 2006; Beaver et al., 2011; Belsky and Beaver, 2011; Althoff et al., 2012; Waldman et al., 2018). Additionally, biologically predisposed traits that are linked to harmful behaviours, such as irritability, have been proposed to only result in aggression prone personality styles when the individual is exposed to early aversive environments (Beauchaine et al., 2009). These findings suggest that biological and environmental factors may combine to influence each other’s pathway to a personality style that is vulnerable to harmful behaviours, such as secondary psychopathy.

### Proximal Processes

#### Emotional Regulation

While personality may cause a predisposition to dual-harm, it is important to consider why an individual may choose to engage in this behaviour. As mentioned, self-regulation may be a distinctive characteristic in those with self-harm and aggressive behaviours (Tang et al., 2013; Richmond-Rakerd et al., 2019). Therefore, it could be that emotional dysregulation theories of self-harm may be extended to dual-harm. In the context of dual-harm, self-harm and aggression may be utilised interchangeably as an emotional dysregulation response to negative emotions. This may occur through over-regulation, in which an individual suppresses their emotional experience by engaging in dual-harm to provide themselves with more perceived tolerable emotions and sensations. Alternatively, emotional dysregulation may occur through under-regulation, in which the individual is unable to use the necessary regulation strategies to sufficiently contain their intense emotional experiences and control their behaviour. Consequently, they fail to inhibit impulsive behaviours and to engage in goal-directed behaviour, thereby leading to dual-harm.

Emotional dysregulation has been implicated in the relationship between psychopathy and harmful behaviours (Long et al., 2014). Therefore, our model suggests that personality constructs that are vulnerable to emotional dysregulation and harmful behaviours, such as secondary psychopathy, may increase an individual’s risk of using dual-harm as an emotional regulation response to their negative emotions. This response may occur through under-regulation or over-regulation of emotional experiences.

#### Interpersonal Functions

In certain situations, it may be that dual-harm serves an interpersonal function. As previously mentioned, harmful behaviours have been found to be influenced by social contextual factors and motivated by interpersonal reasons, such as establishing autonomy or responding to grievance. Furthermore, previous research has demonstrated that self-harm and aggression are associated with increased reactivity to socially adverse situations and impaired value-based decision-making (Coccaro et al., 2007; Richard-Devantoy et al., 2014; Olié et al., 2015). Such impairments have especially been found in personality styles prone to harmful behaviours, including psychopathy. For example, von Borries et al. (2012) found that compared to controls, individuals with psychopathy showed lack of avoidance in response to social threat and were more reactive to such stimuli. Due to the characteristics of secondary psychopathy, such as emotional reactivity, individuals may be more likely to engage in self-harm and aggression in response to distressing social contexts in order to fulfil an interpersonal function. Consequently, our model suggests that, in addition to being utilised as an emotional regulation strategy, dual-harm may also be recognised as a response to interpersonal motivations.

#### Situation/Social Context

While individual related risk-factors, such as personality, are emphasised within our cognitive-emotional model, it is also important to consider the influence of situational and social contextual factors. Situational stressors, including social threat, institutional factors (e.g., crowding and poor management in prisons), family disputes, and bullying, have been shown to be associated with self-harm and aggression (Webb, 2002; Gadon et al., 2006). Given such evidence, researchers have argued that harmful behaviours do not take place in a “vacuum of internal drives and motivations” (Johnstone and Cooke, 2010, p. 182). Rather, situational determinants play an important role. In line with social-cognitive (Hasking et al., 2017) and diathesis-stress theories (Ferguson et al., 2008), we propose that personality traits, such as impulsivity and emotional reactivity in secondary psychopathy, may be triggered into action by an emotionally strenuous situation or social context. The greater the distress caused by this proximal stressor, the more likely the individual is to respond with harmful behaviours.

#### Expectancies

There is a lack of literature that has investigated why individuals may engage in self-harm at one point and aggression at another. Sahlin et al.’s (2017b) study found that self-harm and aggression had a bidirectional relationship. To account for this, the authors suggested that rather than there being a causal unidirectional relationship between self-harm and aggression, where one behaviour leads to another, these behaviours may develop from common vulnerabilities. Therefore, in the context of dual-harm, it may be that individuals engage in self-harm and aggression interchangeably to fulfil a shared function (e.g., emotional regulation or interpersonal motivations).

The behaviour that an individual chooses to engage in at one point in time may be dependent upon the specific situation they are in and their expectations. The above notion is outlined by Hasking et al.’s (2017) cognitive-emotion model which highlights the importance of outcome expectancies and self-efficacy expectancies in NSSI. Outcome expectancies are the expected consequences of a behaviour. Individuals may be more likely to engage in and repeat a behaviour that is linked to positive outcomes (e.g., emotional relief, social attention). Meanwhile, self-efficacy expectancy is an individual’s belief in their ability to successfully carry out the behaviour in a particular context. For example, an offender may believe that the outcome expectancy of self-harm is more positive than that of aggression as they are less likely to receive punishment. Consequently, they may be more likely to self-harm as a way to regulate their emotions. The above notion may account for the proportionally higher rates of self-harm amongst male prisoners in comparison to males within the community (Fazel et al., 2016). Self-efficacy and positive expectations regarding the outcome, emotional regulation and interpersonal functions of harmful behaviours has been found to be associated with increased self-harm and aggression (Smithmyer et al., 2000; Pornari and Wood, 2010; Hasking, 2017; Brausch and Muehlenkamp, 2018; Dawkins et al., 2019a, b, c). Such findings may provide evidence for the influence of expectancies on harmful behaviours.

The role of expectancies may be further highlighted by Daffern and Howells (2009) study of mental health inpatients. Those who engaged in dual-harm tended to perpetrate aggression before self-harm. Moreover, the likelihood of self-harm increased during later stages of the inpatients’ hospital stay. The authors accounted for such findings by suggesting that individuals may have learned over time that aggression does not function well for its expected purpose in the acute mental health ward environment. Accordingly, they include or change to other behaviours, such as self-harm, which may be more appropriate to their particular context. Nijman and à Campo’s (2002) study of dual-harm in mental health inpatients revealed that certain situational factors were distinctively associated with self-harm and aggression. For example, self-harm was more likely to occur in the evening and in private within the patient’s room. These findings were attributed to the notion that patients believe they will be more likely to successfully self-harm during the evening when there are no activities in place and they are able to retreat in the privacy of their rooms where no one can stop them (Nijman and à Campo, 2002).

In light of the above research, our cognitive-emotional model suggests that in the context of dual-harm, self-harm and aggression may be used interchangeably to serve the same purpose (e.g., emotional regulation or interpersonal goals). The specific behaviour the individual chooses to engage in and its function could be influenced by situational determinants and outcome and self-efficacy expectancies. Such expectancies may account for why not all individuals with secondary psychopathy will dual-harm. In that, if an individual has negative self-efficacy or outcome expectancies about self-harm and aggression, they may be less likely to dual-harm as a way to regulate their emotions or for interpersonal reasons (Hasking et al., 2017).

### Feedback Processes

The association between harmful behaviours and personality styles, such as secondary psychopathy, has been argued to be maintained by maladaptive knowledge structures, or cognitive schemas (Anderson and Bushman, 2002; Crawford and Wright, 2007; Gilbert and Daffern, 2011). Schemas are interconnected patterns of thoughts, beliefs, behaviours, and affective states regarding various phenomena (Anderson and Bushman, 2002). These guide cognitive processes and responses to situations (Anderson and Bushman, 2002). Maladaptive schemas are suggested to be formulated and reinforced through repeated exposure to aversive experiences, which give the individual the capability to engage in harmful behaviours. This notion is in line with the Interpersonal Psychological Theory of Suicide (Joiner, 2007; Van Orden et al., 2010) which proposes that the desire and capability to self-harm occurs due to consistent exposure to painful and fearful experiences, such as child abuse. Such exposure leads to an enhanced tolerance to pain and a decreased fear of death or bodily harm. These experiences may also increase the likelihood of aggression, in which witnessing or being a victim of violence increases the capability to engage in aggression (DeWall et al., 2011).

The Schematic Appraisals Model of Suicide (Johnson et al., 2008) further highlights how schema may lead to harmful behaviours. This model suggests that activation of suicide schema leads to thoughts and plans of engaging in suicide as an escape strategy (Johnson et al., 2008). Suicidal schemas may strengthen through repeated experience of volatile emotional states. Moreover, suicidal schemas are suggested to drive, and be reinforced by, situational appraisals (e.g., perception of poor social support) and self-appraisals (e.g., negative perceptions of personal attributes and abilities). This suggests that schemas may influence and be influenced by an individual’s expectancies regarding harmful behaviours.

Young et al.’s (2003) cognitive theory highlights the role of maladaptive schemas in harmful behaviours and personality. The researchers propose that maladaptive schemas develop from a combination of negative childhood experiences and temperamental disposition. Harmful behaviours in personalities, such as psychopathy, are said to mainly be a result of ineffective coping responses to such schemas (Chakhssi et al., 2014a). Research has provided evidence for the association between maladaptive schemas, such as a hostile perception of the world, lack of self-control and low tolerance to frustration, and secondary psychopathy in those with a history of aggression (Chakhssi et al., 2014a).

Our cognitive-emotional model draws from the above theories, the GAM and social-cognitive theories of harmful behaviours to highlight the effect of maladaptive schemas on dual-harm. We suggest that an individual’s adverse experiences, including witnessing or engaging in harmful behaviours, may increase their likelihood of dual-harm through the reinforcement of maladaptive schemas. The above proposal is supported by research that has demonstrated a strong association between perpetrating and/or being a victim of aggression with later self-harm (*odds ratio* = 3.68) (Jordan and Samuelson, 2016; Daukantaitë et al., 2019). Furthermore, studies have found that past self-harm behaviour is the strongest predictor of future self-harm (Beghi et al., 2013). Considering the great likelihood of exposure to aggression within prisons and forensic mental health services, the above notion may account for the high prevalence of dual-harm within forensic settings (Slade, 2018). Moreover, akin to the GAM, our model highlights that when an individual engages in dual-harm, the environmental response and their experience may feed back into their personality, thereby reinforcing their maladaptive schemas. In line with the Interpersonal Theory of Suicide and Schematic Appraisals Model of Suicide, we suggest that such schemas may also influence self-efficacy and outcome expectations regarding harmful behaviours. The above feedback processes increase the risk of endured dual-harm by reinforcing and repeating the model’s process.

In accordance with the Interpersonal Theory of Suicide (Van Orden et al., 2010), within a dual-harm context, individuals may be more likely to engage in NSSI and less severe methods of self-harm at earlier stages as they have not yet acquired the capability to engage in more harmful self-harm behaviours. Due to feedback processes that reinforce and increase tolerance to harmful behaviours, an individual’s capability for more severe methods of self-harm (e.g., overdose and self-immolation) may increase the more they engage in this behaviour. Hence, it may be that the greater the frequency and severity of an individual’s dual-harm, the more likely they are to engage in suicidal behaviour, rather than NSSI, over time, as well as more severe self-harm methods. The above notion may be supported by research that has consistently found NSSI to be a risk-factor for suicidal behaviour and that severity of self-harm positively predicts future suicidal behaviour (Young et al., 2003; Beckman et al., 2018; Olfson et al., 2018; Knorr et al., 2019). Moreover, researchers have suggested that those with emotional regulation impairments gain the capability and desire to engage in suicidal behaviours through the repeated use of other risky behaviours, such as NSSI and aggression (Law et al., 2015). Hence, our model suggests that the frequency and severity of past harmful behaviours perpetuates and reinforces the cycle of dual-harm.

### Model Summary

To summarise, our cognitive-emotional model divides the causal pathways to dual-harm into two main processes: distal and proximal. In the distal processes, biological factors combine with adverse environmental factors to develop a personality style that may predispose an individual to harmful behaviours. In this paper, we focus on secondary psychopathy, though our theory may extend to other personality styles. Through its effects on cognition, arousal and affect, personality traits facilitate the proximal processes of dual-harm by predisposing the individual to both self-harm and aggression, as well as emotional and interpersonal liability, such as emotional dysregulation and emotional reactivity. Consequently, the individual is more likely to engage in dual-harm as an emotional regulation response to their distressing negative emotions. Alternatively, they may engage in dual-harm to serve an interpersonal function. As such, dual-harm may be perceived as a response to internal threat (i.e., regulating intense negative emotions) or external threat (e.g., creating boundaries or communicating distress). The social context and situation the individual is in and their expectancies regarding harmful behaviours combine to lead to the specific function and behaviour they choose to engage in. Finally, the outcome behaviour impacts the individual’s experience and environmental response. This response reinforces maladaptive schemas and feeds back to their personality and expectancies through learning processes, thereby repeating and reinforcing the process of dual-harm.

## Discussion

Our cognitive-emotional model may provide various implications for the development of clinical interventions that aim to target dual-harm. The characteristics in secondary psychopathy have been understood to be developed due to emotional adaption to negative early experiences (e.g., abuse or neglect), and these are perceived to be amenable to treatment (Skeem et al., 2007). Hence, in accordance with our model, interventions aiming to target these traits (e.g., impulsivity, emotional distress, and low behavioural control), as well as emotional dysregulation, may reduce dual-harm in individuals with secondary psychopathy and other related personality styles, such as BPD. This idea may be supported by findings that interventions targeting emotional dysregulation and maladaptive characteristics are effective in reducing the occurrence of self-harm and aggression, including in those with BPD (Castillo et al., 2013; Gratz et al., 2014).

As highlighted by our model, schemas may perpetuate the cycle of dual-harm by reinforcing maladaptive traits and expectancies, leading to repeated engagement with this behaviour. Maladaptive schemas have been shown to be amenable with treatment (Giesen-Bloo et al., 2006; Farrell et al., 2009). For example, schema therapy has been associated with improvements in schemas and traits associated with harmful behaviours, as well as reducing risky behaviours in psychopathy (Chakhssi et al., 2014b). It may be that interventions addressing maladaptive schemas and positive expectations regarding harmful behaviours could reduce dual-harm.

The cognitive-emotional model presented in this report may highlight the potentially limited effectiveness of current strategies in addressing dual-harm. Clinical (e.g., mental health inpatient settings) and forensic (e.g., forensic mental health settings and prisons) services tend to conceptualise and respond to self-harm and aggression separately. Aggression is mostly perceived as unreasonable behaviour and the key focus of responses is to protect other individuals. Consequently, strategies of management tend to be reactive, typically in the form of punishment, restraint or seclusion (Slade, 2019). Conversely, self-harm is perceived as an indication of distress and is treated with care and compassion, with the aim of understanding the individual’s behaviour (Slade, 2019). Our model suggests that, in the context of dual-harm, individuals may use self-harm and aggression interchangeably to fulfil the same function. Therefore, by managing self-harm and aggression separately, services may inadequately recognise the co-occurrence, potential interchangeability and shared function of these behaviours in those who dual-harm.

Previous research has shown that reactive strategies of violence management within clinical and forensic settings, for example placing offenders on a basic regime or solitary confinement, increase the risk of future antisocial behaviour and self-harm (Duxbury and Whittington, 2005; Kenning et al., 2010; Kaba et al., 2014). Our cognitive-emotional model may account for such findings. The model suggests that an individual’s negative experience in response to their dual-harm (e.g., punishment and containment management strategies) strengthens their maladaptive schema and expectancies, thereby reinforcing dual-harm behaviour. This may lead to a coercive cycle of harmful behaviour-aversive response, in which harmful behaviours are met with an aversive response, which in turn, leads to future harmful behaviours, and so on. Despite the potential risk associated with using reactive approaches with those who dual-harm, offenders who dual-harm have been reported to spend a longer time in prison and twice as much time in segregation and other restrictive programmes compared to offenders who engage in aggression alone (Slade, 2019). Such findings suggest that current management approaches may be ineffective in reducing self-harm and aggression in those who dual-harm, and could in fact increase their risk of these behaviours.

Individuals who dual-harm represent a high-risk group, and control and punishment-orientated strategies are often necessary to protect others from dangerous situations posed by these individuals. Consequently, those who dual-harm may not be able to access certain interventions due to the risk they present to others (Slade, 2019). To address this, it may be important for services to adopt an integrated approach that considers an individual’s history of both aggression and self-harm on a case-by-case basis, as well as the risk they pose to themselves and others. This may allow the development of more effective risk-assessment, intervention and management strategies that are tailored to the individual’s particular risk profile. Evaluating the effectiveness of different management strategies on a case-by-case basis may also allow better identification of approaches that are in the best interest of the individual. This may help break the suggested harmful behaviour-aversive response cycle by taking into account the duality of an individual’s harmful behaviour and utilising strategies that inhibit the reinforcement of both their self-harm and aggression. To guide such evaluations, future research should aim to investigate the effect of various management approaches on dual-harm.

The inadequate research investigating dual-harm is reflected in the lack of guidance for the effective care of those who engage in this behaviour. Harmful behaviours are systematically perceived and managed separately from the top (e.g., distinct government areas and policies) to ground level (e.g., society, healthcare, and forensic services). Nevertheless, this paper and previous research highlight that such a separation between self-harm and aggression may be insufficient, and even unhelpful. Our work supports a shift from exclusively approaching self-harm and aggression separately, to considering these behaviours together in the context of dual-harm and the possible unique needs of those who engage in this behaviour. To implement such a major shift, it is necessary to adapt our perceptions of harmful behaviours as a potential false dichotomy, to a more unified construct. This may be achieved by expanding the literature on the aetiology, function, and characteristics of dual-harm. Our cognitive-emotional model informs such research by providing various testable hypotheses regarding the causal pathways and motivations of this behaviour. These include the association between personality and dual-harm, and the role of emotional regulation, distal biological and environmental factors, situational factors, expectancies, and feedback processes in this behaviour.

## Limitations

It is important to note the limitations of our cognitive-emotional model. Dual-harm is yet to be established as a separate, clinically valid construct, and it is unclear how this behaviour should be meaningfully defined. There is a need for research that tests the hypotheses presented by our model and investigates the impact of adopting various definitions of this behaviour. Moreover, the model we have presented draws from our narrative review of the literature of harmful behaviours. While a systematic review was beyond the scope of this paper, future systematic reviews should be conducted that evaluate previous research in-depth to provide evidence for the various components of our theory.

In this article, we have considered self-harm more broadly by not distinguishing between suicidal and non-suicidal behaviours, or different methods of self-harm. Additionally, research has shown an association between self-harm/suicidal ideation and aggression (Hill et al., 2020; Koyama et al., 2020). It may be important to differentiate between NSSI, suicidal behaviours and ideation in order to identify their distinct and shared causal mechanisms in dual-harm.

While the cognitive-emotional model includes distal biological factors, it does not consider the potential role of proximal biological mechanisms. This is because our theory intended to focus on the evidence-based cognitive and emotional aspects of harmful behaviours. There is currently a lack of evidence that supports the inclusion of proximal biological mechanisms as a central component of dual-harm. Nevertheless, there may be important biological factors that contribute to this behaviour. For example, research has implicated impairments in prefrontal areas of the brain in emotional dysregulation (Beauchaine and Cicchetti, 2019). Therefore, this factor could play a role in those who may dual-harm as an emotional dysregulation response. Research should investigate the role of biological factors in dual-harm to provide a more comprehensive biopsychosocial theory of this behaviour.

Finally, the theory proposed in this report is not intended to be an exhaustive account of dual-harm. Given the early stage in which the literature of dual-harm is currently in, we did not intend to provide a comprehensive model that includes all potential causal pathways. Our model may not generalise to all who dual-harm, and not everyone who engages in this behaviour will do so for emotional regulation and interpersonal reasons or have a personality style that is vulnerable to harmful behaviours. Future research should test the proposed model and other theories of dual-harm across different groups of individuals, including those with secondary psychopathy, BPD diagnoses and other personality styles, in order to assess its generalisability.

## Conclusion

To the best of our knowledge, our cognitive-emotional model provides one of the only theoretical frameworks for dual-harm. This work encourages research and practice to move toward an integrated approach that considers the duality of self-harm and aggression and the possibility that these behaviours may have common causal pathways in the context of dual-harm. To achieve this, there is a need for robust research that will help us better understand, predict and treat this behaviour. Our model provides various hypotheses that can be tested with such research. Further investigations could establish dual-harm as a unique construct, that should be understood as being separate from sole-harm behaviour. This may help us address challenges in current policy and practice by facilitating the development of more integrated and focused assessment, management and treatment strategies for dual-harm.

## Data Availability Statement

The original contributions presented in the study are included in the article/supplementary material, further inquiries can be directed to the corresponding author/s.

## Author Contributions

MS wrote the manuscript. AF, DP, and PJT contributed to manuscript revision, read, and approved the submitted version. All authors contributed to the article and approved the submitted version.

## Conflict of Interest

The authors declare that the research was conducted in the absence of any commercial or financial relationships that could be construed as a potential conflict of interest.
